# Parenting Style and Adolescent Mental Health: The Chain Mediating Effects of Self-Esteem and Psychological Inflexibility

**DOI:** 10.3389/fpsyg.2021.738170

**Published:** 2021-10-13

**Authors:** Biao Peng, Ningning Hu, Huiying Yu, Hanshi Xiao, Jie Luo

**Affiliations:** ^1^School of Public Administration, Hunan Normal University, Changsha, China; ^2^Nursing School, Xinjiang Medical University, Ürümqi, China; ^3^School of Marxism, Guizhou Medical University, Guiyang, China; ^4^School of Psychology, Guizhou Normal University, Guiyang, China

**Keywords:** parenting style, mental health, self-esteem, psychological inflexibility, mediating model, Chinese adolescents

## Abstract

Based on interpersonal acceptance–rejection theory, family systems theory, and psychological inflexibility theory, this study aimed to examine the mediating roles of a protective factor (self-esteem) and a risk factor (psychological inflexibility) on the influence of parenting style on adolescent mental health. A sample of Chinese adolescents (*n* = 916, 46% male, mean age = 14.44 years, *SD* = 1.84 years) completed the Short Egna Minnen Barndoms Uppfostran, the Rosenberg Self-esteem Scale, the Avoidance and Fusion Questionnaire for Youth, the Satisfaction with Life Scale, and the Depression Subscale of the Youth Self-Report. Results show that the self-esteem and psychological inflexibility play a chain mediating role in the relationship between parenting style and adolescent mental health. Specifically, parental emotional warmth had a positive effect on adolescent mental health through the chain mediating effects of self-esteem and psychological inflexibility. Parental rejection and parental over-protection had negative effects on adolescent mental health by lowering self-esteem but increasing psychological inflexibility. These results provide further guidance in the prevention of and intervention in adolescent mental health problems.

## Introduction

Adolescence is a critical period of rapid physical and mental development. A blocked development process will often cause adolescents to experience psychological crises and a variety of mental health problems (Baumrind, 1991). Poor mental health is the leading cause of disability in young people, accounting for a large proportion of the global disease burden faced by adolescents, with long-term impacts. Moreover, poor mental health in adolescence is one factor that influences risk-taking behaviors (e.g., self-harm, use of tobacco, alcohol and drugs), risky sexual behaviors, and exposure to violence. The ongoing effects of such behaviors can be persistent and have serious implications throughout life (World Health Organization, 2020).

In recent years, an increasing number of countries and organizations have highlighted the importance of adolescent mental health awareness. The World Health Organization (WHO) has launched the WHO Special Initiative for Mental Health (2019–2023), highlighting the need for universal health coverage to include mental health (World Health Organization, 2019) and recommending new WHO guidelines on how to promote mental health awareness among adolescents (World Health Organization, 2020). The Chinese government has implemented the Healthy China Action Plan (2019–2030) and the Healthy China Action Plan—Children and Adolescents' Mental Health Plan (2019–2022; National Health Commission of the People's Republic of China, 2019a,b). It is clearly of great important to focus on further studies into adolescent mental health.

The mental health of adolescents is influenced by many factors. Personal factors (e.g., biological and psychological characteristic factors) and environmental factors (e.g., family, school and peer group) are considered to be the main factors which affect adolescent mental health (Carr, 2015). Indeed, the family factor (i.e., parenting style) has been shown to be one of the most important factors affecting adolescent mental health (Newman et al., 2008). Parenting style not only directly affects the mental health of adolescents, but also has been shown to have a lasting impact on the development of adolescents' personality and other psychological characteristics (Rohner and Britner, 2002; Rohner et al., 2005; Huang et al., 2010). Therefore, it is necessary to examine the influence mechanism of parenting style on adolescent mental health. This study thus focused on the roles of self-esteem and psychological inflexibility on the relationship between parenting style and adolescent mental health in order to provide targeted guidance for the prevention of and intervention in adolescent mental health problem.

### Parenting Style and Mental Health

Parenting style is defined as a set of attitudes a parent holds toward their child that are communicated to the child and that, taken together, create an emotional climate in which the parent's behaviors are expressed (Darling and Steinberg, 1993). According to Baumrind (1971), parenting style is divided into authoritarian, authoritative, and permissive parenting.

Both the interpersonal acceptance-rejection (IPAR) theory (i.e., the new development of parental acceptance-rejection theory) and family systems theory indicate that parenting style has an effect on adolescent mental health. The IPAR theory proposes that across cultures and other sociodemographic groups, interpersonal acceptance and rejection consistently predict the psychological and behavioral adjustment of children and adults (Rohner, 2016; Rohner and Lansford, 2017). Parents are generally major attachment figures for children, and parental acceptance and rejection have an extremely important effects on children's mental health. Specifically, parental emotional warmth, as a positive parenting style, has a positive effect on adolescent mental health; meanwhile, parental rejection, as a negative parenting style, has a negative and persistent impact on adolescent mental health (Rohner and Britner, 2002; Rohner et al., 2005; Rohner, 2016; Rohner and Lansford, 2017). According to family systems theory, meanwhile, the stability, harmony and health of the whole family system have a crucial influence on children's psychological and emotional growth. The parent–child relationship is an important subsystem of the family system. Parenting style exerts a crucial influence on the growth of a child's mental health through the interactions of family functions (Bowen and Kerr, 2009). Cross-cultural comparative study and meta-analysis have supported the above two theories (Khaleque, 2013; Wang et al., 2018; Rohner et al., 2019, 2020). Although many theoretical and empirical studies have shown that parenting style affects adolescent mental health, few studies have considered the roles of both protective and risk factors. Previous studies have suggested that self-esteem and psychological inflexibility are closely related to parenting style (Mann et al., 2004; Kashdan and Rottenberg, 2010). More specifically, self-esteem has been shown to be an important protective factor in mental health (Mann et al., 2004; Moksnes and Reidunsdatter, 2019), while psychological inflexibility has been shown to be a risk factor in mental health (Kashdan and Rottenberg, 2010). As such, we hypothesized that both are highly likely to play mediating roles in the relationship between parenting style and adolescent mental health.

### Mediating Role of Self-Esteem

Self-esteem is defined as the combined set of one's thoughts and feelings about their own worth and importance (Rosenberg, 1965). It is the evaluative and emotional dimension of the self-concept and is considered to be equivalent to self-respect, self-assessment, and self-worth (Baumeister, 1997; Harter, 1999). Vulnerability models suggest that self-esteem and stress will interact to produce psychopathology such that high self-esteem buffers individuals from the deleterious consequences of stress, whereas low self-esteem increases their vulnerability to the effects of stress (Zeigler-Hill, 2011; Moksnes and Reidunsdatter, 2019). This theory is supported by a large number of studies, which show that self-esteem is positively correlated with physical and mental health, positive self-esteem has an important protective effect on adolescents' mental health, while low self-esteem has a negative effect on adolescents' mental health and overall life satisfaction (Abe, 2004; Mann et al., 2004; Boden et al., 2008; Gao et al., 2015; Moksnes and Reidunsdatter, 2019; Pazzaglia et al., 2020).

Parenting style, self-esteem, and mental health are all significantly correlated. According to IPAR and family systems theory, a negative parenting style, which might include behavior such as parental rejection or over-protection, can damage a child's self-esteem, leading to low self-esteem and low mental health (Herz and Gullone, 1999; DeHart et al., 2006; Raboteg-Saric and Sakic, 2014; Rohner and Lansford, 2017; Perez-Gramaje et al., 2020). Meanwhile, a positive parenting style, which might include behavior such as parental emotional warmth, has been shown to be conducive to the child's development of self-esteem, leading to high self-esteem and high mental health (Robertson and Simons, 1989; Qian and Xiao, 1998; Rohner et al., 2005; Bowen and Kerr, 2009; Perez-Gramaje et al., 2020; Szkody et al., 2020). Self-esteem mediates the relationship between parental bonding and general mental health (Yamawaki et al., 2011). Therefore, the self-esteem might be a mediating variable between parenting style and adolescent mental health.

### Mediating Role of Psychological Inflexibility

Psychological inflexibility (also called psychological rigidity) is an important concept in Acceptance and Commitment Therapy (ACT), and is the opposite of psychological flexibility. Hayes et al. (2006) defined psychological inflexibility as a set of behaviors that pertain to an individual's diminished ability to fully experience the present moment and adapt their behavior to achieve their goals. Psychological inflexibility has been identified as a root cause of human suffering and maladaptive functioning (Bond et al., 2011; Hayes et al., 2011). At present, empirical research has shown that psychological inflexibility is an extremely important predictor of mental health (Fledderus et al., 2010; Levin et al., 2014; Lucas and Moore, 2020).

Psychological inflexibility stems out of six processes: inflexible attention, disruption of chosen values, inaction or impulsivity, attachment to a conceptualized self, cognitive fusion, and experiential avoidance (Hayes et al., 2006). These six processes are formed on the basis of relational frames. According to ACT, relational framing under poor contextual control makes it difficult for humans to maintain flexible, focused, and voluntary attention on their present experience. In other words, the relational frames formed by poor contextual control lead to psychological inflexibility (Hayes et al., 2011). Based on this, we hypothesized that a negative parenting style combined with poor contextual control may lead to adolescent psychological inflexibility.

Previous studies have indicated that poor parenting environments lead to inflexible and experiential avoidance of self-regulatory strategies (Rosenthal et al., 2006; Morris et al., 2007). A 6-year longitudinal study showed that low warmth and high control parenting styles both predicted low psychological flexibility (high psychological inflexibility); meanwhile high warmth parenting styles predicted low psychological inflexibility in adolescents (Williams et al., 2012). We therefore hypothesized that parenting style may be a predictor of psychological inflexibility, for example parental emotional warmth negatively predicting psychological inflexibility, while parental rejection and over-protection positively predicting psychological inflexibility. We expected that psychological inflexibility would be highly likely to play a mediating role between parenting style and adolescent mental health.

### The Relationship Between Self-Esteem and Psychological Inflexibility

Both psychological inflexibility and self-esteem have been shown to be significant predictors of mental health (Al-Jabari, 2012), and may play mediating roles between parenting style and adolescent mental health (Yamawaki et al., 2011; Williams et al., 2012). To date, very few studies have focused directly on the relationship between self-esteem and psychological inflexibility, and their mediating impacts on the effect of parenting style on adolescent mental health. However, we are able to infer the relationship between these two variables based on relevant theories and research results.

Self-esteem is the evaluative and emotional dimension of self-concept, and is considered to be one of the most important elements of self-concept (Baumeister, 1997; Harter, 1999), as well as existing at the highest level of self-concept (Judge and Bono, 2001). People with low self-esteem have a negative self-concept (Blaine and Crocker, 1993). According to ACT, self-concept and conceptualized self are similar concepts, as they both emphasize the self-experience (Hayes et al., 2011). Additionally, the conceptualized self, which is a form of negative self-experience and self-knowledge, can have a negative effect on psychological flexibility (e.g., increasing psychological inflexibility; Hayes et al., 2011). Moreover, one's attachment to the conceptualized self plays a role in the formation of psychological inflexibility (Hayes et al., 2011). A recent study has shown that self-esteem can negatively predict psychological inflexibility, and psychological inflexibility plays a mediating role in self-esteem and eating disorders in adolescents (Koushiou et al., 2020). Psychological inflexibility thus could be a mediating variable between self-esteem and mental health, with parenting style also having an impact on adolescent mental health through chain mediation between self-esteem and psychological inflexibility.

### The Present Study

Although previous studies have examined the relationship between the four variables of parenting style, self-esteem, psychological inflexibility, and mental health, the role of self-esteem and psychological inflexibility specifically in the impact of parenting style on mental health has not yet been examined. The current study aimed to explore the mediating roles of self-esteem and psychological inflexibility on the effects of parenting style on adolescent mental health. The current study was guided by the following hypotheses:

H1: Self-esteem plays a mediating role in the relationship between parenting style and mental health.
H2: Psychological inflexibility might be a mediator in the relationship between parenting style and mental health.
H3: Self-esteem and psychological inflexibility together play a chain mediating role in the relationship between parenting style and mental health.

## Materials and Methods

### Participants

The participants involved in this study were recruited from three middle schools in Zhejiang, Hunan, and Guizhou provinces, China. A total of 916 students were recruited to complete the questionnaires. Participants were aged 11–19 years old (*M* = 14.44, *SD* = 1.84), 421 (46.0%) were boys and 495 (54.0%) were girls, 224 (24.5%) were in 7th grade, 196 (21.4%) were in 8th grade, 82 (9.0%) were in 9th grade, 148 (16.2%) were in 10th grade, 185 (20.2%) were in 11th grade, and 81 (8.8%) were in 12th grade.

### Procedure

Before beginning the investigation, we consulted with the local education departments and investigating schools to obtain their approval to perform the study. The questionnaires were completed in a classroom setting while participants were attending their regular classes. Participants provided written consent prior to completing the study questionnaires, having been informed of the purpose, confidentiality, and anonymity of this study. This study involving human participants was reviewed and approved by Guizhou Medical University.

### Measurements

#### The Short Egna Minnen Barndoms Uppfostran (S-EMBU)

The S-EMBU (Arrindell et al., 1999) is a short form of the EMBU (Perris et al., 1980), developed to measure parental rearing behavior. The S-EMBU includes three dimensions: rejection, emotional warmth, and over-protection. Each item of the S-EMBU adopts a four-point Likert scale that ranges from 1 (No, never) to 4 (Yes, most of the time). The Chinese version of S-EMBU has been validated for use in Chinese populations (Jiang et al., 2010). In this study, the Cronbach α coefficients of the subscales of paternal emotional warmth, maternal emotional warmth, paternal rejection, maternal rejection, paternal over-protection, and maternal over-protection were 0.89, 0.88, 0.85, 0.84, 0.73, and 0.74, respectively.

#### The Rosenberg Self-Esteem Scale (RSES)

The RSES (Rosenberg, 1965) was developed to assess the individual's level of self-esteem. The Chinese version of the Rosenberg Self-esteem Scale has been validated for use in Chinese adolescents (Wei et al., 2018). The Cronbach alpha coefficient of the scale in this study was 0.90.

#### The Avoidance and Fusion Questionnaire for Youth (AFQY)

The AFQY (Greco et al., 2008) was developed to measure psychological inflexibility. The Chinese version of the AFQY (AFQ-Y8) was revised by Chen et al. (2019) and validated for use in Chinese populations, and contains eight items measured using a five-point scale. The higher the total score, the higher one's level of psychological inflexibility. In this study, the Cronbach's α of the AFQ-Y8 was 0.82.

### Mental Health

According to Greenspoon and Saklofske (2001), the dual-factor model of mental health (DFM) combines a positive health indicator (e.g., subjective well-being) and a traditional negative illness indicator (e.g., psychopathology) to comprehensively measure mental health. This study used the two-factor mental health model (Hai et al., 2015) which includes the two subscales of life satisfaction and depression. The Satisfaction With Life Scale (SWLS, Diener et al., 1985) consists of five items measured using a seven-point Likert scale. The Chinese version of the SWLS was validated for use in Chinese adolescents (Yue et al., 2006). The Cronbach's α of the scale in this study was 0.77. The depression subscale of the Youth Self-Rating index (YSR, Achenbach, 1991) includes 16 items measured using a three-point Likert scale, and has been validated for use in Chinese adolescents (Liu et al., 1997). The Cronbach's α of the subscale in the present study was 0.93.

### Data Analysis Strategy

Statistical analyses were performed using SPSS 24.0 and AMOS 23.0 for IBM. First, we used the Harman single factor test (Podsakoff et al., 2003) to test for common method bias (CMB). Second, descriptive statistics and Pearson's product-moment correlations were calculated. Considering that gender and grade could have been additional influencing factors on adolescent mental health, we used gender and grade as independent variables and mental health as the dependent variable to carry out independent sample *t*-tests and one-way analysis of variance. We decided to control these variables specifically based on the results of the aforementioned analysis. Third, a structural equation model (SEM) was used to examine the mediating effects. We used the following indicators to evaluate the model fit: comparative fit index (CFI), Tucker-Lewis Index (TLI), and root mean square error of approximation (RMSEA). According to Hu and Bentler (1999), a good model fit is characterized by RMSEA values below 0.05 with CFI and TLI scores above 0.95, whereas RMSEA values smaller than 0.08 with CFI and TLI scores above 0.90 indicate an adequate model fit. Finally, the bootstrap method was also used to test the mediating effect of the model.

## Results

### Common Method Bias Test (CMB)

Exploratory factor analysis resulted in 18 factors with eigenvalues greater than 1. The first factor accounted for 26.27% of total variance, which is less than 40% of the critical standard, which indicated that common method bias was not apparent.

### Descriptive Statistics and Significance Test

The mean, standard deviation, and Pearson's product-moment correlations coefficient of each variable were calculated. Parenting style, self-esteem, psychological inflexibility, and mental health (depression, life satisfaction) were shown to be significantly related (see Table 1).

**Table 1:** Means, standard deviations, and correlations among the main variables.

	**1**	**2**	**3**	**4**	**5**	**6**	**7**	**8**	**9**	**10**
1. PEW	1									
2. PR	−0.51	1								
3. POP	−0.20	0.58	1							
4. MEW	0.86	−0.43	−0.21	1						
5. MR	−0.46	0.75	0.43	−0.54	1					
6. MOP	−0.22	0.50	0.78	−0.22	0.57	1				
7. SE	0.45	−0.37	−0.27	0.47	−0.36	−0.27	1			
8. PI	−0.30	0.35	0.31	−0.28	0.34	0.35	−0.55	1		
9. LS	0.49	−0.40	−0.27	0.50	−0.41	−0.28	0.54	−0.35	1	
10. Dep	−0.33	0.46	0.40	−0.34	0.45	0.41	−0.65	0.65	−0.48	1
*M*	19.42	8.95	16.71	20.34	9.20	17.77	28.89	21.52	20.27	24.90
*SD*	4.96	3.30	4.08	4.71	3.31	4.23	5.42	6.07	5.48	6.31

*PEW, paternal emotional warmth; MEW, maternal emotional warmth; PR, paternal rejection; MR, maternal rejection; POP, paternal over-protection; MOP, maternal over-protection; SE, self-esteem; PI, psychological inflexibility; LS, life satisfaction; Dep, Depression; all the rs are significant at p < 0.01*.

An independent samples *t*-test analysis indicated that there was a significant difference between males and females in depression (*t* = −2.88, *p* = 0.004, *M*: male, 24.25; female, 25.45), but no significant differences in life satisfaction between genders (*t* = −1.67, *p* = 0.095, *M*: male, 19.94; female, 20.55). One-way ANOVA showed that there were significant grade differences in depression (*F* = 4.68, *p* < 0.001, *M*: 7th, 23.83; 8th, 24.18; 9th, 24.52; 10th, 25.13; 11th, 26.42; 12th, 26.08), but no significant differences in life satisfaction (*t* = 1.45, *p* = 0.205, *M*: 7th, 20.71; 8th, 20.38; 9th, 20.91; 10th, 19.41; 11th, 19.92; 12th, 20.52).

### Mediation of Self-Esteem and Psychological Inflexibility

In line with prior item parceling studies (Landis et al., 2000; Little et al., 2002), we used the “item-to-construct balance” method to parcel items, and the SEM was used to examine the mediating effects of self-esteem and psychological inflexibility between parenting style and psychological inflexibility. Given that there were significant gender and grade differences in depression, these two variables (gender and grade) were used as control variables. After controlling for the effects of gender and grade, we found that the data fitting of the three models was acceptable (see Table 2).

**Table 2:** Model fit statistics for SEM.

**Model**	**χ*^**2**^*/*df***	**CFI**	**TLI**	**RMSEA (90% CI)**
M1 Parental emotional warmth and mental health	5.30	0.95	0.94	0.069 (0.062,0.075)
M2 Parental rejection and mental health	4.51	0.96	0.95	0.062 (0.056,0.068)
M3 Parental over-protection and mental health	4.22	0.96	0.95	0.059 (0.053,0.066)

*CFI, comparative fit index; TLI, Tucker-Lewis index; RMSEA, root mean square error of approximation*.

**Figure 1:**
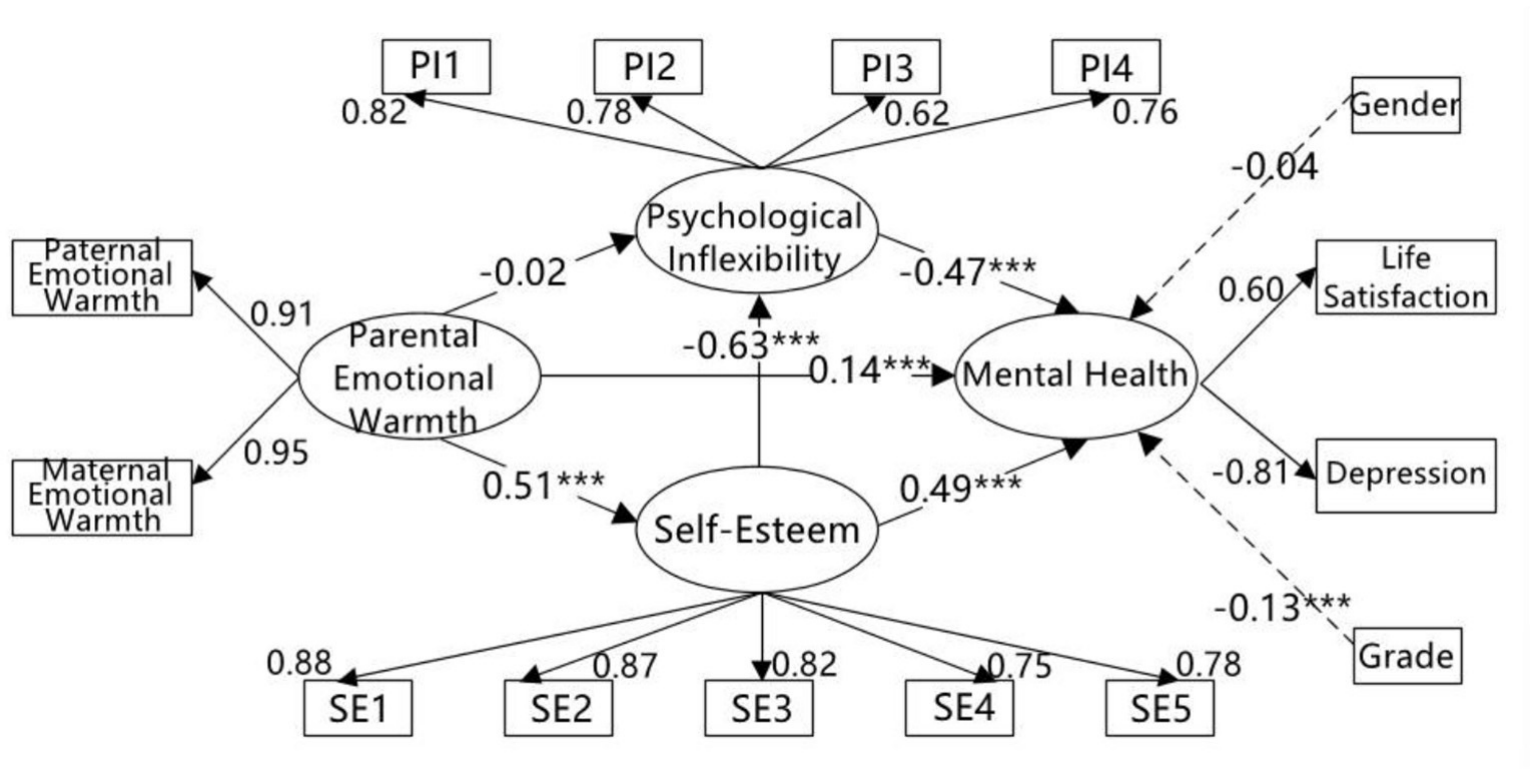
The mediating roles of self-esteem and psychological inflexibility between parental emotional warmth and mental health. PI1–PI4, four parcels of psychological inflexibility; SE1–SE5, five parcels of self-esteem; ^***^p < 0.001.

The mediation model showed that parental emotional warmth had a significant positive direct effect on both self-esteem (β = 0.51, *t* = 15.27, *p* < 0.001) and mental health (β = 0.14, *t* = 4.31, *p* < 0.001), but that the direct effect of parental emotional warmth on psychological inflexibility was not significant (β = −0.02, *t* = −0.60, *p* > 0.05). Meanwhile, self-esteem had a significant negative direct effect on psychological inflexibility (β = −0.63, *t* = −15.58, *p* < 0.001) and a significant positive direct effect on mental health (β = 0.49, *t* = 10.50, *p* < 0.001). The psychological inflexibility had a significant negative direct effect on mental health (β = −0.47, *t* = −10.44, *p* < 0.001; see Figure 1).

**Figure 2:**
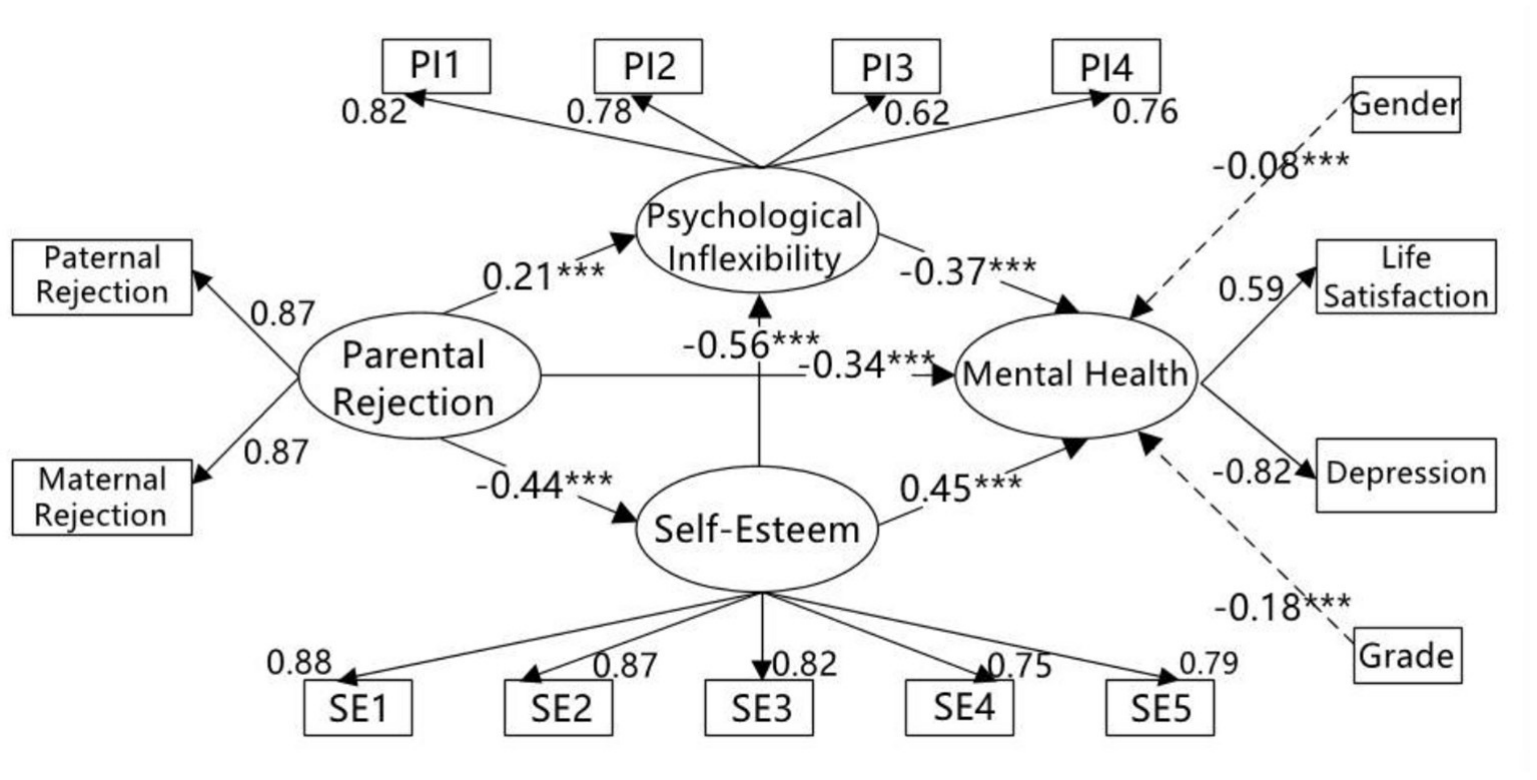
The mediating roles of self-esteem and psychological inflexibility between parental rejection and mental health. PI1–PI4, four parcels of psychological inflexibility; SE1–SE5, five parcels of self-esteem; ^***^p < 0.001.

As shown in Figure 2, parental rejection had a significant negative direct effect on self-esteem (β = −0.44, *t* = −11.92, *p* < 0.001) and mental health (β = −0.34, *t* = −10.07, *p* < 0.001), and a significant positive direct effect on psychological inflexibility (β = 0.21, *t* = 5.72, *p* < 0.001). Self-esteem had a significant negative direct effect on psychological inflexibility (β = −0.56, *t* = −14.81, *p* < 0.001) and had a significant positive direct effect on mental health (β = 0.45, *t* = 11.26, *p* < 0.001). Psychological inflexibility had a significant negative direct effect on mental health (β = −0.37, *t* = −9.02, *p* < 0.001).

**Figure 3:**
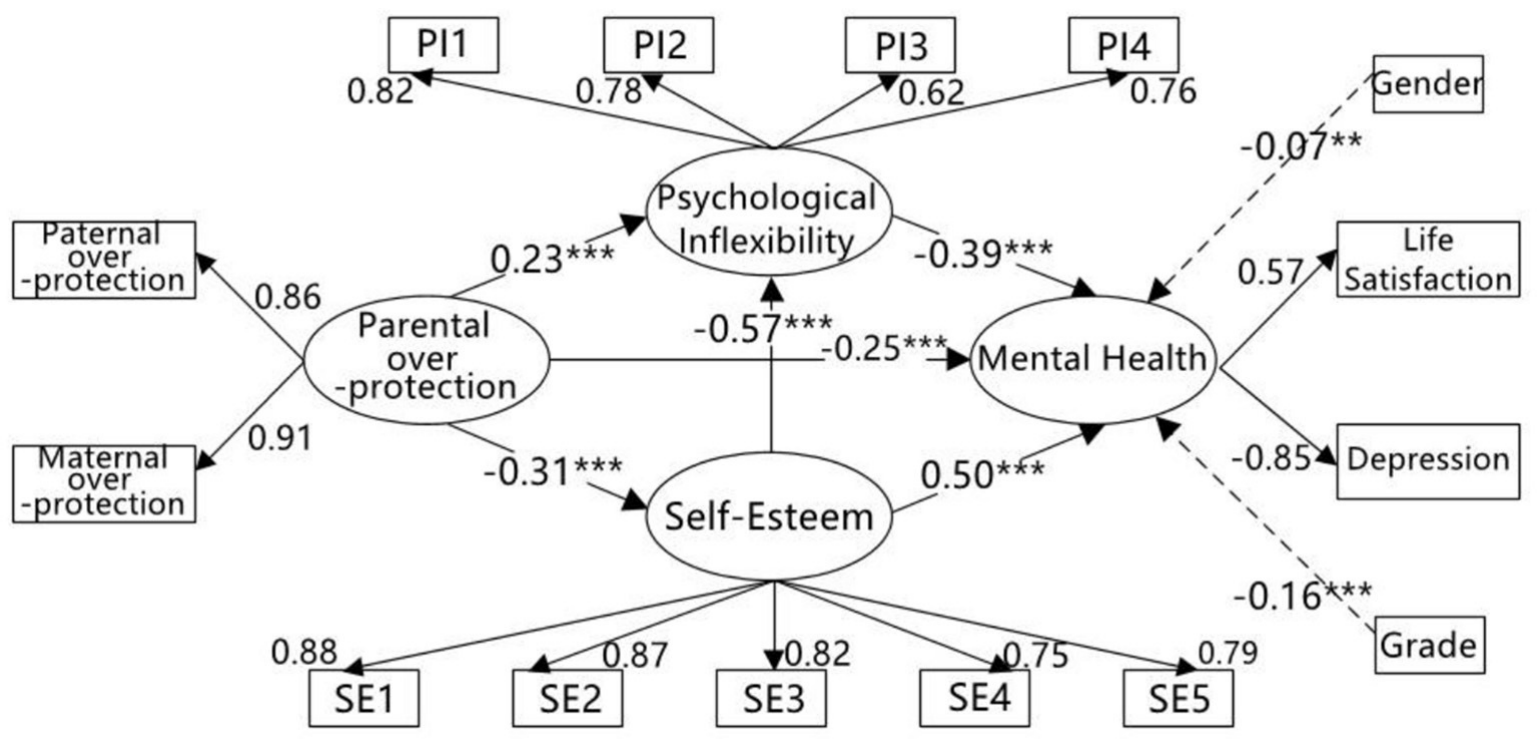
The mediating role of self-esteem and psychological inflexibility between parental over-protection and mental health. PI1–PI4, four parcels of psychological inflexibility; SE1–SE5, five parcels of self-esteem; ^**^p < 0.01; ^***^p < 0.001.

The mediation model further indicated that parental over-protection had a significant negative direct effect on self-esteem (β = −0.31, *t* = −8.56, *p* < 0.001) and mental health (β = −0.25, *t* = −8.04, *p* < 0.001), and had a significant positive direct effect on psychological inflexibility (β = 0.23, *t* = 6.92, *p* < 0.001). Self-esteem had a significant negative direct effect on psychological inflexibility (β = −0.57, *t* = −16.23, *p* < 0.001) and had a significant positive direct effect on mental health (β = 0.50, *t* = 11.73, *p* < 0.001). Psychological inflexibility had a significant negative direct effect on mental health (β = −0.39, *t* = −9.00, *p* < 0.001; see Figure 3).

Finally, to further examine the significance of the mediating effects of self-esteem and psychological inflexibility, we performed a bootstrap analysis using the bias correction nonparametric percentage test to repeat sampling 5,000 times and calculate the 95% CI. The results indicated that self-esteem plays a mediating role in the relationship between parenting style (e.g., parental emotional warmth, rejection and over-protection) and mental health. Meanwhile, psychological inflexibility appears to play a mediating role in the relationship between negative parenting style (e.g., parental rejection and over-protection) and mental health, but psychological inflexibility had no significant mediating effect between positive parenting style (i.e., parental emotional warmth) and mental health. Self-esteem and psychological inflexibility played a chain mediating role in the relationship between parenting style and mental health (see Table 3).

**Table 3:** Bootstrap analysis of the mediating model.

	**Effect**	**Path**	**Standardized β**	**The size of effects**	**95% CI**
Model 1	Total	PEW→ MH	0.55		
	Direct	PEW→ MH	0.14		
	Indirect1	PEW→ SE→ MH	0.25	45.45%	0.13, 0.24
	Indirect2	PEW→ PI→ MH	0.01	1.82%	−0.02, 0.03
	Indirect3	PEW→ SE→ PI→ MH	0.15	27.27%	0.08, 0.14
Model 2	Total	PR→ MH	−0.70		
	Direct	PR→ MH	−0.34		
	Indirect1	PR→ SE→ MH	−0.20	28.57%	−0.30, −0.16
	Indirect2	PR→ PI→ MH	−0.08	11.43%	−0.13, −0.05
	Indirect3	PR→ SE→ PI→ MH	−0.09	12.86%	−0.14, −0.07
Model 3	Total	POP→ MH	−0.56		
	Direct	POP→ MH	−0.25		
	Indirect1	POP→ SE→ MH	−0.16	28.57%	−0.20, −0.09
	Indirect2	POP→ PI→ MH	−0.09	16.07%	−0.11, −0.05
	Indirect3	POP→ SE→ PI→ MH	−0.07	12.50%	−0.09, −0.04

*PEW, parental emotional warmth; PR, parental rejection; POP, parental over-protection; PI, psychology inflexibility; SE, self-esteem; MH, mental health*.

## Discussion

The purpose of this study was to explore the influence of parenting style on adolescent mental health with consideration of a protective factor (self-esteem) and a risk factor (psychological inflexibility). In line with IPAR theory, family systems theory, and psychological inflexibility theory, our results support the idea that self-esteem plays a mediating role in the relationship between parenting style and mental health, while psychological inflexibility plays a mediating role in the relationship between negative parenting style and mental health. Furthermore, self-esteem and psychological inflexibility play a chain mediating role between parenting style and mental health.

First, similar to findings from previous studies (Herz and Gullone, 1999; DeHart et al., 2006; Yamawaki et al., 2011; Gao et al., 2015; Szkody et al., 2020), the current study supports the theory that parenting style can significantly predict levels of self-esteem (i.e., parental warmth positively predicts self-esteem while parental rejection or parental overprotection negatively predict self-esteem), as well as the proposition that self-esteem plays a partial mediating role in the relationship between parenting style (e.g., parental emotional warmth, rejection, over-protection) and adolescent mental health (Hypothesis 1). Self-esteem is the evaluation and emotional dimension of self-concept (Baumeister, 1997; Harter, 1999). Parental rearing behaviors could play a crucial role in shaping a child's self-esteem (Shaffer and Kipp, 2013). A negative parenting style tends to cause children to hold a low sense of self-evaluation, while a positive parenting style leads to children forming a high sense of self-evaluation. At the same time, self-esteem is also an important protective factor in adolescent mental health (Abe, 2004; Mann et al., 2004; Boden et al., 2008; Zeigler-Hill, 2011). According to IPAR theory, a continuous negative influence of parental rejection on children and adolescents results in to the loss of self-esteem, the formation of negative personality qualities, and negative impacts on adolescent mental health. Meanwhile, parental acceptance (i.e., emotional warmth) contributes to the development of self-esteem, the formation of healthy personality traits, and the improvement of adolescent mental health (Rohner et al., 2005; Khaleque, 2013; Rohner, 2016; Rohner and Lansford, 2017). According to the family systems theory, parental over-protection can lead to a hierarchical parent-child relationship that reduces an adolescent's ability to make their own decisions and self-evaluate, and instead increases their feelings of worthlessness and insecurity while negatively affecting self-esteem, which in turn predicts their mental health (Bowen and Kerr, 2009).

Second, psychological inflexibility plays a partial mediating role in the relationship between negative parenting style (i.e., parental rejection, and over-protection) and mental health. Our results are consistent with findings by Williams et al. (2012) which show that a negative parenting style can predict psychological inflexibility. Psychological inflexibility, which is the opposite of psychological flexibility and linked to basic human processes (Hayes et al., 2011), has been shown to be a root cause of human suffering and maladaptive functioning. The psychological inflexibility model is simultaneously a model of psychopathology, of psychological health, and of psychological intervention (Hayes et al., 2011). The predictive effect of psychological inflexibility on depression, anxiety, and other mental health problems has been confirmed in previous studies (Churchill et al., 2013; Dionne et al., 2013; Panayiotou et al., 2014). Relational frame theory and functional contextualism propose that relational frames are not only conducive to problem solving, but also contribute to rigid rule-following and experiential avoidance. Relational framing is considered a foundation of the formation of psychological inflexibility in that relational framing under poor contextual control will lead to psychological inflexibility (Hayes, 2004). The family environment is an important context in which children and adolescents form their relational frames. Adolescents who have been rejected and overprotected by their parents often form unhelpful or harmful relational frames based on contextual cues (e.g., “I'm unlovable”) and adopt pliancy in their relational frames, leading to further psychological inflexibility, which is believed to be the root cause of psychological disorders (Hayes et al., 2011).

It should be pointed out that, while ACT as a theory has pointed out that poor context will lead to psychological inflexibility, it does not clearly indicate that a positive context can reduce psychological inflexibility (i.e., improve psychological flexibility). However, the current study found a significant negative correlation between parental emotional warmth and psychological inflexibility, suggesting that parental emotional warmth as a positive context can negatively predict psychological inflexibility, which is consistent with research by Williams et al. (2012). However, the current study indicated that psychological inflexibility had no significant mediating effect on the model of parental emotional warmth on mental health. In the modeling the effect of parental emotional warmth on mental health, the effect of parental emotional warmth on psychological inflexibility was completely mediated by self-esteem. According to ACT theory, this could be because the negative effect of parental emotional warmth on psychological inflexibility is achieved entirely through the increase of self-esteem. Parental emotional warmth can enhance self-esteem, thereby breaking the limits a child has of their conceptualized self, thereby reducing their level of psychological inflexibility (Hayes et al., 2011).

Third, the current study showed that self-esteem positively predicts psychological inflexibility, which is in line with the findings of Koushiou et al. (2020). The current study also indicated that self-esteem and psychological inflexibility play a chain mediation role in the relationship between parenting style and mental health (Hypothesis 3). According to PAR and family systems theory, parenting style is the key to the formation of self-esteem. A positive parenting style is conducive to the development of self-esteem, while a negative parenting style is not conducive to the development of self-esteem (Rohner et al., 2005; Bowen and Kerr, 2009). Self-esteem, as the evaluative and emotional dimension of self-concept, is at the highest level of self-concept (Baumeister, 1997; Harter, 1999; Judge and Bono, 2001). Individuals with lower self-esteem might have more a negative self-concept (Blaine and Crocker, 1993). According to ACT theory, self-concept is the core of verbal and cognitive processing and is similar to the concept of conceptualized self. The conceptualized self is the direct by-product of training in naming, categorization, and evaluation (Hayes et al., 2011). It is like a spider's web that contains all the categories, interpretations, evaluations, and expectations associated with the self. Constraining it can lead to psychological inflexibility (Hayes et al., 2011). Furthermore, attachment to the conceptualized self is considered to be one of the six processes of mental ossification (Hayes et al., 2011). People with low self-esteem might be more inclined to follow the rules of low self-evaluation (i.e., pliancy) and more inclined to attach themselves to the conceptualized self (i.e., the more negative conceptualized self), which might lead to psychological inflexibility (Hayes et al., 2011). Psychological inflexibility has been considered to be the root cause of many mental disorders (Hayes et al., 2011), and has been empirically supported as an extremely important indicator for the prediction of mental health (Levin et al., 2014; Lucas and Moore, 2020). It follows, then, that parenting style can predict self-esteem, and self-esteem can predict mental health through its negative predictive effect on psychological inflexibility.

## Implications

This study explored the effects of parenting style on adolescent mental health through the perspectives of a protective factor (self-esteem) and a risk factor (psychological inflexibility), and provides theoretical and practical guidance for families and schools in how to proactively support adolescent mental health. Our findings suggest that self-esteem plays an important role in the influence of parenting style on adolescent mental health. Parents should cultivate their children's self-esteem through demonstrations of high emotional warmth, low rejection, and low over-protection behaviors. Our findings also suggest that psychological inflexibility plays an important role not only in the influence of parenting style on adolescent mental health, but also in the influence of self-esteem on adolescent mental health. Therefore, parents should not only pay attention to the cultivation of children's self-esteem, but also focus on children's psychological flexibility. Parents should cultivate their children's self-esteem and psychological flexibility through high emotional warmth, low rejection and low over-protection, and then improve their children's mental health. Specifically, parents should express love, care, affection, nurturance and emotional support to their children through physical, verbal or symbolic means, avoid expressing indifference, aggression, neglect and rejection to their children, and avoid excessive control over their children. In addition, studies have shown that parental psychological flexibility will predict the parenting atmosphere and parent-child relationship (Brassell et al., 2016). Parents should learn some ACT techniques, such as acceptance, cognitive defusion, flexible attention to the present moment, mindfulness, self-as-context, values, and committed action, in order to enhance parental psychological flexibility. Improve family atmosphere and parent–child relationship. At the same time, these techniques can be taught to children to reduce their level of psychological inflexibility and promote mental health.

## Limitations and Future Direction

The findings from the current study should be considered in light of its limitations. First, this study only investigated middle school students from Mainland China, so it may not be possible to generalize the results to adolescents in other geographic areas or cultural contexts; future research should attempt to replicate our findings in populations of other cultural backgrounds (i.e., Western samples). Second, although this study explored the mediating role of self-esteem and psychological inflexibility in the relationship between parental style and mental health using a cross-sectional study; a longitudinal investigation should be considered for future research. Finally, psychological inflexibility models have received extensive attention in the field of mental health, but until now little research exists into the specific relationship between self-esteem and psychological inflexibility. The current study has explored the predictive effect of self-esteem on psychological inflexibility, and the chain mediating effect of self-esteem and psychological inflexibility on the relationship between parenting style and adolescent mental health. But whether reducing an adolescent's level of psychological inflexibility (i.e., increasing mental flexibility) will increase their self-esteem is not clear; future research should focus on the effects of psychological flexibility on self-esteem.

In general, this study has contributed to the understanding of the mechanism by which parenting style influences adolescent mental health. Parental rearing behavior may predict adolescent mental health through the mediating role of self-esteem and psychological inflexibility.

## Data Availability Statement

The datasets supporting the conclusions of this article will be made available by the first author and corresponding authors.

## Ethics Statement

The studies involving human participants were reviewed and approved by Guizhou Medical University. Written informed consent to participate in this study was provided by the participants' legal guardian/next of kin.

## Author Contributions

BP, JL, and HY designed the study protocol. BP performed the statistical analysis and drafted the manuscript. JL guided the statistical analysis, interpretation of the results, and edited the final manuscript. HY provided financial support, as well as guided the first draft of the paper. NH completed the literature review and participated in the study design and interpretation analysis. HX provided guidance on the overall design of the study and the revision of the manuscript. All authors contributed, read, and approved the final manuscript.

## Funding

This research was supported by the National Social Science Foundation Youth Project of China (20CKS059) and Guizhou Philosophy and Social Science Planning Key Project (21GZZD51).

## Conflict of Interest

The authors declare that the research was conducted in the absence of any commercial or financial relationships that could be construed as a potential conflict of interest.

## Publisher's Note

All claims expressed in this article are solely those of the authors and do not necessarily represent those of their affiliated organizations, or those of the publisher, the editors and the reviewers. Any product that may be evaluated in this article, or claim that may be made by its manufacturer, is not guaranteed or endorsed by the publisher.
